# Structure, *Z′* = 2 Crystal Packing Features of 3-(2-Chlorobenzylidene)-5-(*p*-tolyl)furan-2(3*H*)-one

**DOI:** 10.3390/molecules26082137

**Published:** 2021-04-08

**Authors:** Vyacheslav S. Grinev, Oksana A. Mayorova, Tatyana V. Anis’kova, Alexandra S. Tikhomolova, Alevtina Yu. Yegorova

**Affiliations:** 1 Institute of Biochemistry and Physiology of Plants and Microorganisms RAS, Prospekt Entuziastov 13, 410049 Saratov, Russia; beloousova011@yandex.ru; 2 Institute of Chemistry, N.G. Chernyshevsky Saratov National Research State University, Ulitsa Astrakhanskaya 83, 410012 Saratov, Russia; aniskovatv@mail.ru (T.V.A.); bondartsova.alexandra@ya.ru (A.S.T.); yegorovaay@gmail.com (A.Y.Y.)

**Keywords:** crystal structure, crystallographically independent forms, rotamers, quantum chemical modeling, rotational barrier, 3-arylidene-5-arylfuran2(3*H*)-one

## Abstract

3-(2-Chlorobenzylidene)-5-(*p*-tolyl)furan-2(3*H*)-one (**1**), C_18_H_13_ClO_2_, crystallizes with *Z* = 8 and *Z′* = 2, and the structure at 100 K has orthorhombic (*Pna*2_1_) symmetry. Each kind of molecule takes part in π–π stacking interactions to form infinite chains parallel to the *c* axis. We believe that the existence of two forms can be explained by the probable rotation around a single C–C bond. The quantum chemical modeling reveals that these molecules are almost equivalent energetically, and they can be described as the two most stable conformers (rotamers) with a minor rotational barrier of about 0.67 kcal/mol.

## 1. Introduction

Much attention is paid to the study of the biological active compounds containing both furan-2(3*H*)-one and substituted benzene rings linked via “spacers” of various length and rigidity, which are capable of adopting different conformations adapting to receptors [1,2,3,4,5,6,7,8]. There are several ways to obtain such compounds using 5-aryl substituted furan-2(3*H*)-ones as initial substrates. Among them there is an azo coupling reaction of aryldiazonium salts based on the substituted anilines giving rise to arylazohydrazones of furan-2(3*H*)-ones with an azo group as a spacer demonstrating anti-inflammatory and analgesic activity [9,10,11,12,13,14,15].

Another approach to obtain structural information on hydrazones with a shorter spacer is a condensation of substituted aromatic aldehydes with furan-2(3*H*)-ones resulting in corresponding arylmethylidene derivatives. Both hydrazones and arylmethylidene derivatives of furan-2(3*H*)-ones are of interest for studying their *Z*/*E*-tautomeric equilibrium and conformational preferences. Arylmethylidene (arylidene) derivatives of furan-2(3*H*)-ones are also structural fragments of various biologically active compounds. The 2-(2-nitrobenzylidene) malonate and some related structures were synthesized and studied as a novel group of TLR4 signaling inhibitors to reveal the mechanism of their inhibitory effect [16]. Recently, some structural, spectroscopic, and photophysical properties of alkylamino substituted 2-arylidene and 2,5-diarylidene cyclopentanone dyes were studied [17]. Arylmethylidene derivatives are also available as scaffolds for the synthesis of various heterocyclic compounds of different complexity. These substances combine the properties of unsaturated carbonyl compounds and esters, allowing the implementation of reactions with various C-nucleophiles such as acetoacetic ester [18], acetylacetone [19], cyclohexanone [20], and mono- and *N*,*N*-binucleophiles [21,22]. Owing to the presence of a conjugated double bond, arylidene derivatives can easily add azides [23,24] oxygen (to give rise corresponding epoxides [25]), and halogen [26]. Many of the studied reactions are stereoselective, and the configuration of the resultant products is associated with the structure of the reagents.

**Figure 1:**
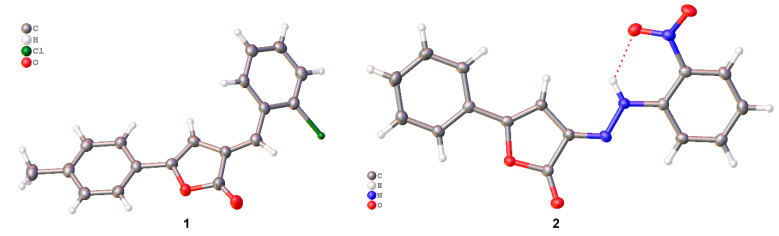
General view of molecules: non-flat arylidene (**1,** current study) and flat hydrazone (**2**) derivatives of furan-2(3*H*)-one in a crystal. Atoms are represented by thermal vibration ellipsoids (*P* = 50%). The H-bond is indicated by a dotted line.

Recently, we reported basic crystal structural features of the arylmethylidene compound **1**, which is one of the representatives of hydrazones, namely (*E*)-3-[2-(2-nitrophenyl)hydrazineylidene]-5-phenylfuran-2(3*H*)-one (**2**, Figure 1). It has been shown that azo coupling with compounds containing an active methylene group leads to the possible existence of several tautomeric forms due to prototropic tautomerism due to the migration of a proton from the C(2) carbon atom to the N(2) nitrogen atom or O(2) oxygen atom. Compound (**2**) exists in the crystal in the *E*-hydrazo tautomeric form, which was confirmed by the corresponding interatomic distances N(1)–N(2) of 1.3437(16) Å and N(1)–C(2) of 1.2984(18) Å.

X-ray diffraction analysis of (**2**) showed that the hydrazone tautomeric form is favorable for the intermolecular H-bond of the corresponding substituents in the aryl fragment since an additional stabilization of the *E*-configuration is possible. The entire molecule lies almost in one plane due to the extended conjugation chain covering the phenyl ring at position 5, the double bond and the lactone carbonyl group in the furanone ring, as well as the 2-nitrophenylhydrazo fragment [5].

3-Arylmethylidene derivatives also have the ability to exist in the *E*- and *Z*-configuration relative to the exocyclic C=C bond. The introduction of a bulky substituent in an *ortho*-position of the benzene ring as well as the closeness of the H-atoms of mentioned benzene ring and at the C-4 atom in furan-2(3*H*)-one moiety may cause some hindrance during the rotation around single bonds to give rise to some stable rotational structures (rotamers), which are bonded by non-covalent π–π stacking interactions in the crystal. The study of the conformational preferences of some *o*-substituted heterocyclic systems is important from the point of view of structure–activity relationships (SAR), because different conformers may have non-equivalent receptor affinities [27,28,29]. For the study of the conformational features of different compounds, a number of techniques have been used, including NMR, UV–Vis, FTIR spectroscopy, X-ray diffraction, and quantum chemical calculations. Intermolecular interactions have been implicated in the formation of high *Z′* structures [30]. Today, scientists pay great attention to crystals with *Z′* ≥ 2. From the one hand, crystals containing more than one independent molecule exhibit lower symmetry [31]. On the other hand, there is an approach based on a topological analysis of the experimental distribution of electron density which allows one to investigate the response of the single molecule in crystals with *Z′* ≥ 2 to the surroundings [32] even caused by the weak interactions [33,34].

In this study, we present the results of X-ray diffraction of the titled compound demonstrating a *Z′* = 2 crystal packing. To reveal some reasons of the existence of a mixture of two forms in the condensed state, we performed DFT modeling. We also suggest that the intrinsic rotation around simple C–C bond is a possible mechanism of the transition of these two forms from one to another and evaluated the intrinsic rotation barrier in the gaseous phase.

## 2. Results and Discussion

### 2.1. Solving Structure Attempts

Our attempts to solve the structure in the higher symmetry space group (including in the centrosymmetric space group *Pnam*) were unsuccessful and failed to establish a reasonable structural model. The ADDSYM (PLATON) program did not indicate any missed symmetry for the structure solved in *Pna*21. Furthermore, the ADDSYM analysis for the structure of (**1**) solved in *P*1 only suggested the centrosymmetric space group *Pna*21. Solving the structure using the SUPERFLIP and SHELXT programs having algorithms for determining the space group gave the same results.

### 2.2. Crystal Structure Analysis

**Figure 2:**
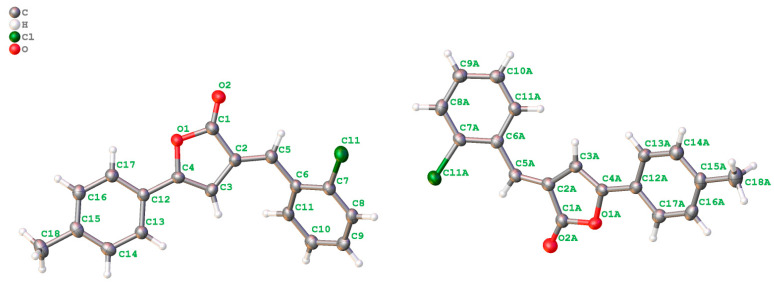
The geometry of the two crystallographically independent forms of (**1**) in the asymmetric unit. Displacement ellipsoids are drawn at the 50% probability level.

The content of the asymmetric unit of (**1**), along with the atom-labeling scheme, is shown on Figure 2. This structure has been deposited in the Cambridge Crystallographic Data Centre with the deposition number CCDC 1456486. These data can be obtained free of charge via https://www.ccdc.cam.ac.uk/structures/ (accessed date 19 March 2021, or from the Cambridge Crystallographic Data Centre, 12, Union Road, Cambridge CB2 1EZ, UK; Fax: +44-1223-336033).

**Figure 3:**
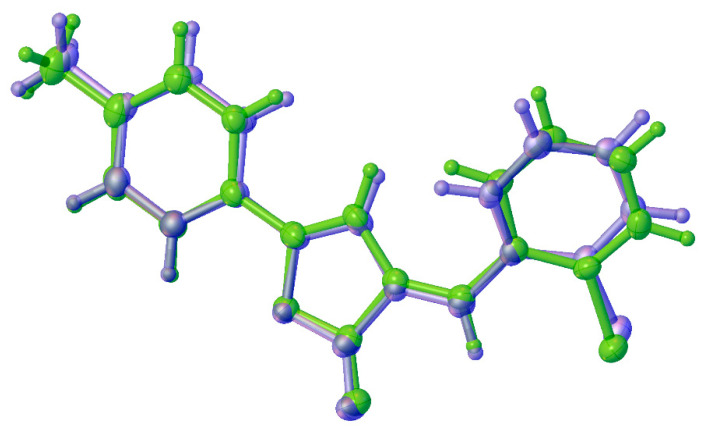
The overlay diagram of the two crystallographically independent forms of **1** in the asymmetric unit. Displacement ellipsoids are drawn at the 50% probability level.

Compound (**1**) crystallizes with *Z* = 8 and *Z′* = 2 in the orthorhombic crystal system, space group *Pna*21. In the asymmetric unit, two crystallographically independent forms are found. These two molecules reveal structural closeness. Most authors determine the *Z′* parameter as the number of symmetry independent molecules [30]. *Z′* > 1 may be formally presented as a co-crystallization where the two components are chemically the same. On the one hand, applying the inversion symmetry operation to one of these forms, one can see a significant similarity in the overlay diagram, especially in the part of molecules containing the flat furan-2(3*H*)-one ring with the *p*-tolyl substituent (Figure 3). On the other hand, these twins can be represented as rotamers of (**1**), because aryl-substituted furan moieties of two forms are very similar, and the most pronounced distinction is the location of the arylmethylidene substituent, which can be illustrated by the values of the C(2)–C(5)–C(6)–C(7) and C(2A)–C(5A)–C(6A)–C(7A) torsion angles which are –144.5(5)° (rotamer **1a**) and 157.6(5)° (rotamer **1b**), respectively. As a result, there are differences in the interatomic distances between chlorine and hydrogen atoms in forms (**1a**) and (**1b**). In (**1a**), the contacts Cl(1)···H(5A) and H(5A)···O(2) are 2.785 and 2.714 Å, respectively, and the distance H(11A)···H(3A) is 2.301 Å. The corresponding distances in (**1b**) are shorter by 0.1–0.2 Å (2.649, 2.629 and 2.150 Å, respectively). The C(5)H···Cl(1)/C(5A)H···Cl(1A) contacts are significantly shorter than the sum of Bondi’s vdw (Van der Waals)radii (in non-bonded contact distances) of 2.95 Å [35], indicating a pronounced electrostatic attraction between chlorine and hydrogen atoms. The O(2)···C(5)H/O(2A)···C(5A)H contacts are close to the sum of the vdw radii (2.72 Å), and in (**1b**), the attraction between hydrogen and oxygen atoms is greater. The C(3)**H**···C(11)**H**/C(3A)**H**···C(11A)**H** contacts [2.30118(8)/2.14979(7) Å] are significantly shorter than the sum of the vdw radii (2.38–2.40 Å), especially for the **1b** twin, which indicates a repulsion between hydrogen atoms leading to the distortion of planarity of the whole molecule (**1**) in the single crystal and we note that short H···H distances often play an important role in defining molecular conformation and crystal packing [36].

The molecule of (**1**) is structurally related to the recently described 3-[2-(2-nitrophenyl)hydrazono]-5-phenylfuran-2(3*H*)-one (**2**) [5]. Both compounds (**1**) and (**2**) include 5-arylfuran-2(3*H*)-one moieties and differ in the substituents at position 3. 

According to the position of the substituents and rings towards the C(2)–C(5) double bond, the molecules of (**1**) are *E*-isomers. It was known that the arylidene derivatives of five-membered heterocycles may be *Z*-configurational isomers as well as in the *E*-form at the exocyclic C=C double bond. This mostly depends not on the steric volume of a substituent but on the ability of the substituent to form non-covalent interactions within the molecule, as well as between neighboring molecules [37,38]. The presence of the wide network of the hydrogen bonds in the crystal and the non-covalent interaction between the H-atom of the methoxy group and the π-system of the other substituent allows one to stabilize the *E*-form of 3-phenyl-4-(2,4,6-trimethoxybenzylidene)isoxazol-5(4*H*)-one, while the corresponding 3-phenyl-4-(2,4,6-trimethylbenzylidene)isoxazol-5(4*H*)-one is in the *Z*-form as the more common configuration of such arylidene substituted molecules in the solid state [37].

The *p*-tolyl substituent at position 5 and the furan-2(3*H*)-one ring in (**1**) are in one plane, as they also are in (**2**). In contrast, in 3-phenyl-4-arylidene-isoxazol-5(4*H*)-ones, the 3-phenylisoxazolone fragments are non-coplanar [37]. Hydrazone (**2**) is almost flat owing to the presence of an extended conjugation along the whole molecule. In the furanone ring of (**2**), the heteroatom O(1) is coming out of the plane, and the C(2)–C(3)–C(4)–O(1) torsion angle is 1.25(14)°. The furanone ring of (**1**) is more flat, the deviations from the plane of the ring of atoms are minimal, and the absolute values of the corresponding C(2)–C(3)–C(4)–O(1) torsion angle are less than 1°. The 2-nitrophenyl substituent slightly deviates from the plane of the furanone ring, and the N(1)–N(2)–C(11)–C(12) torsion angle is 8.08(17)°. At the same time, the molecule of (**1**) is non-planar, and the arylmethylidene substituent is characterized by the C(2)–C(5)–C(6)–C(7)/C(2A)–C(5A)–C(6A)–C(7A) torsion angles of –144.5(5)° and 157.6(5)°, respectively, which are greater than the corresponding angle of –18.41(18)° [the O(3)–N(3)–C(16)–C(11) torsion angle] in (**2**). This is probably due to the more pronounced flexibility of the arylhydrazone substituent, as well as to the longer distance between the nitroaromatic and furanone fragments in (**2**), while the arylmethylidene substituent is located closer to the furanone ring in (**1**). An additional factor that does not permit the most advantageous position for conjugation, in which all the aromatic rings of the molecule would be in one plane, is the presence of the bulky chlorine atom as the substituent in the *ortho* position of the benzene ring. Nevertheless, the interatomic distances C(1)–C(2)/C(1A)–C(2A) of 1.480(6)/1.477(6) Å and C(5)–C(6)/C(5A)–C(6A) of 1.461(6)/1.451(6) Å are significantly shorter than the corresponding distances in alkanes (typically about 1.541 ± 0.003 Å) and are close to the average single bond length of conjugated olefins (about 1.466 ± 0.005 Å), which shows their partial double-bond character. At the same time, the interatomic distances C(2)–C(5)/C(2A)–C(5A) of 1.354/1.351 Å are slightly longer than the corresponding average value of the isolated double bond in olefins (about 1.335 ± 0.002 Å) and they are of partly single-bonded character. The interatomic distances C(1)–O(2)/C(1A)–O(2A) with their values of 1.201(5)/1.202(5) Å are typical of an average C=O bond length of 1.200 Å [39]. These facts point to the presence of conjugation in the molecules of (**1**). Moreover, in system (**2**) there is an intramolecular hydrogen bond between the hydrogen atom at N(2) and the O(3) atom of the nitro group, which is absent from structure (**1**). 

### 2.3. Packing and π–π Stacking Interactions

**Figure 4:**
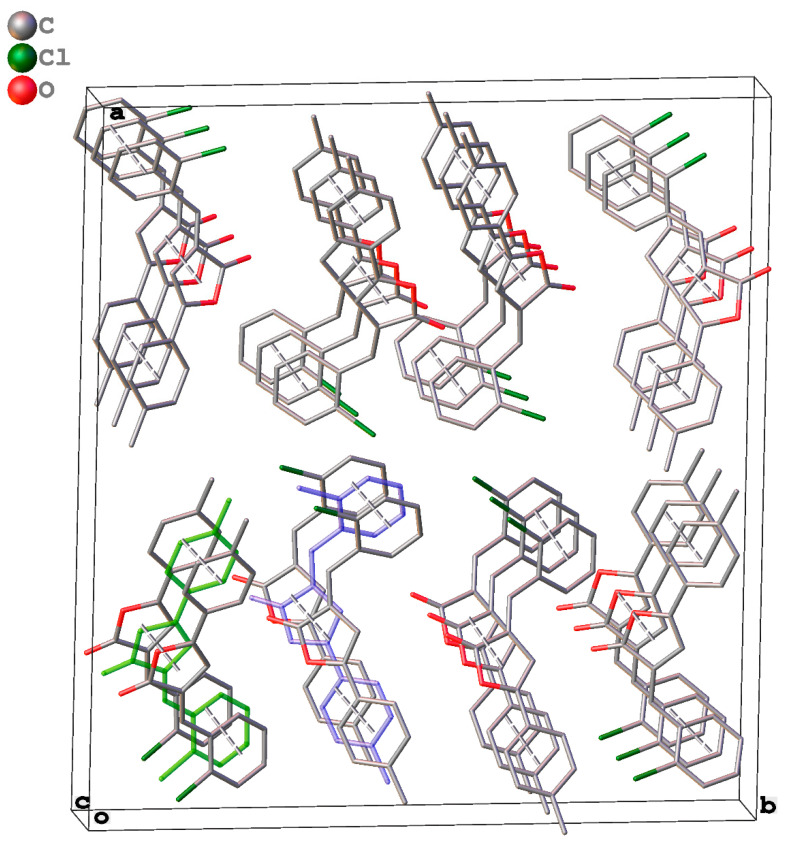
Extended packing diagram of the stacked molecules of (**1**), showing non-covalent interactions by dotted cylinders resulting in infinite chains parallel to the *c* axis. Hydrogen atoms omitted for clarity. One (**1a**) molecules is marked blue, and one (**1b**) molecule is marked green.

The unit cell volume of (**1**) [2776.3(3) Å^3^] is relatively large for this type of molecule [1373.71(3) Å^3^ for the unit cell of (**2**)]. The cell unit of (**1**) contains four pairs of rotamers, whereas the unit cell of (**2**) contains only four molecules. In the projection along the *c* axis, it can be seen that the molecules are arranged in a checkerboard pattern—each form, (**1a**) and/or (**1b**), is surrounded by the molecules of another one from four sides (Figure 4). The molecules **1a** only interacting with symmetry-related **1a** molecules (and similarly for **1b**). Considering the packing of the molecules along the *a* axis, one may note that each rotamer is arranged with another form head-to-head or tail-to-tail.

Figure 4 is an extended packing diagram showing that the rotamers of each kind are linked into infinite chains parallel to the *c* axis via π–π stacking interactions between the chlorophenyl rings, the benzene rings of the *p*-tolyl substituents at position 5, and furan-2(3*H*)-one rings. The intercentroid distances between all rings of each type are identical and are 3.8037(2) Å for both rotamers. Such parameters of stacking interactions as interplanar distances, ring offsets, and angles θ for all three types of rings differ not only among themselves within each form but also between the same rings of the other type. The major parameters of π–π stacking interactions are presented in Table 1.

The smallest intercentroid distance and the maximum angle θ are observed in the case of the *p*-tolyl substituents of (**1a**). The maximal interplanar distance and accordingly, the minimal angle θ, were observed in the *o*-chlorobenzene rings of (**1b**). In general, the values of the interplanar distances are in the range of 3.3803(7)–3.519(3) Å, and the angles θ are in the range of 22.33(8)–27.31(4)°, which is typical of stacked carbo- and heterocyclic molecules, which are relatively small and almost flat [40,41,42,43].

**Figure 5:**
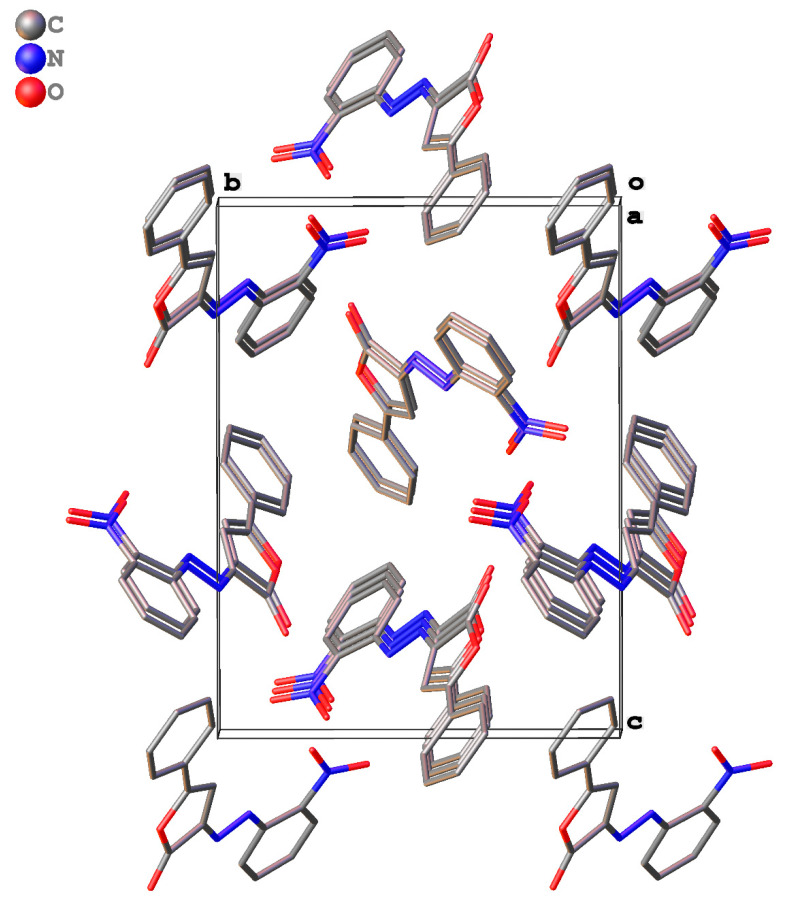
Extended packing diagram of molecules of (**2**), view along the *a* axis. Hydrogen atoms omitted for clarity. Centroid–centroid distance of the benzene and furan-2(3*H*)-one rings of 7.47720(10) Å and ring offset of 6.675(2) Å indicate no non-covalent interactions between molecules.

In contrast to the non-flat arylmethylidene (**1**), the planar hydrazone (**2**) shows only intramolecular H-bonds in a crystal as non-covalent interactions and reveals no π–π stacking (Figure 5).

### 2.4. Theoretical Investigation of the Rotational Barrier

**Figure 6:**
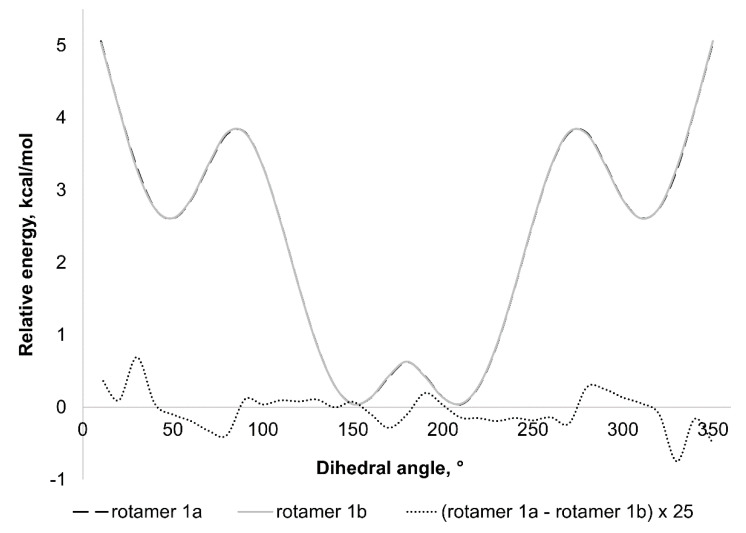
Relative energy profiles for the rotation around the C(5)–C(6)/C(5A)–C(6A) bond in rotamers (**1a**) and (**1b**). The difference between relative energies of the two rotamers multiplied by 25 is shown by the dotted line.

The existence of two types of molecules, (**1a**) and (**1b**), in the crystal state can be illustrated by the rotation around the single C–C bond. The similarity of the aryl-substituted furan moieties of two forms suggests that the rotation is a likely reason for the presence of two forms of (**1**), which we call rotamers. To quantify the rotational barrier energy in (**1**), we performed the quantum chemical calculations of both rotamers in vacuum, separately at the B3LYP/6-311++G(d,p) level of theory. The curve expressing the dependence of the relative values of the system’s energy on the value of the C(2)–C(5)–C(6)–C(7) torsion angle is symmetric and there are two global minima, two local minima, two local maxima, and one global maximum (Figure 6). All important contacts in the extrema of the energy rotational curves of rotamers (**1a**) and (**1b**) are in Table 2.

The structures of both rotamers found in the crystal of (**1**) demonstrate good correlation with corresponding models (a comparison of geometrical parameters are listed in Table 3) and correspond to the two global minima in the relative energy curve (Figure 6) with calculated values for the dihedral angles at these minima of –152.303° and 152.251° for (**1a**) and (**1b**), respectively. The most energetically disadvantageous structure represents a rotamer with the torsion angle of about 0°. This structure is completely flat, despite the fact that a maximum conjugation of multiple bonds should be expected for such structure as a stabilization factor. The flat configuration of the molecule is not possible due to the C(5)H···C(11)H/C(5A)H···C(11A)H close contacts of about 2.07 Å, as well as the C(3)H···Cl(1)/C(3A)H···Cl(1A) distances of about 2.50 Å, which are significantly shorter than the corresponding sums of the vdw radii, illustrating strong repulsion between these atoms. The local maxima corresponded to torsion angles of about ±90°. In this configuration, all important contacts are greater than the corresponding sums of the vdw radii, but the structure exhibits no conjugation of the furanone ring with the arylmethylene fragment and this affects the total energy of the system. The difference between the energies of (**1a**) or (**1b**), as compared with their optimized structures, is about 3.88 kcal/mol. The two local minima between local and global maxima correspond to the structures with torsion angles of about ±50°. The rotational barrier, calculated as the difference between the energies of the global maximum and global minimum, is about 5.66 kcal/mol. The transition of rotamer (**1a**) to (**1b**) and back is possible through a rotation around the C(5)–C(6)/C(5A)–C(6A) single bond by approximately 50°, with transfer to the second flat state, corresponding to the local maximum, halfway through the rotation. This rotamer is characterized by C(3)H···C(11)H/C(3A)H···C(11A)H distances of about 1.97 Å and C(5)H···Cl(1)/C(5A)H···Cl(1A) contacts of about 2.54 Å, close contacts, which are smaller than the corresponding sums of the vdw radii. These distances are similar to those in the most disadvantageous structure. The barrier of this transition as the difference between the energies of the local maximum structure and the global minimum state is only about 0.67 kcal/mol.

## 3. Material and Methods

### 3.1. Physical Measurements

The ^1^H (400 MHz) and ^13^C NMR (100 MHz) spectra in acetone-*d*_6_ were recorded with a Varian (Agilent) 400 spectrometer (Agilent Technologies, Santa Clara, CA, USA), internal standard was tetramethylsilane (TMS). Chemical shifts (δ) are reported in ppm. Elemental analysis was performed on a CHNS analyzer “Elementar Vario MICRO cube” (Elementar Analysensysteme GmbH, Hanau, Germany). The melting point was determined on a Boetius table. The progress of the reaction and the purity of the synthesized compound were monitored by TLC on ALUGRAM^®^ SIL G UV_254_ plates (MACHEREY-NAGEL GmbH & Co. KG, Düren, Germany), and a hexane–ethyl acetate–acetone (2:2:1) mixture was the eluent.

### 3.2. Synthesis, Characterization, and Crystallization

**Scheme 1:**
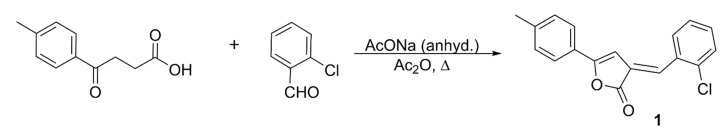
Synthesis of the titled compound (**1**).

The 3-(2-chlorobenzylidene)-5-(*p*-tolyl)furan-2(3*H*)-one (**1**) was obtained by the procedure described in [40] (Scheme 1):

A mixture of 1 g (5.2 mmol) of 4-oxo-4(*p*-tolyl)butanoic acid, 0.73 g (5.2 mmol) of 2-chlorobenzaldehyde, and 0.43 g (5.2 mmol) of anhydrous sodium acetate was heated in 12 mL of acetic anhydride for 45 min. After being cooled, the reaction mixture was poured into water, and the precipitate of (I) was filtered, recrystallized from 96% ethanol, and dried. Yellow crystals (aq. EtOH) yield 1.17 g (76%), mp 151–153 °C; ^1^H NMR (400 MHz, acetone): δ 2.40 (s, 3H, Me), 6.70 (s, 1H, Fu). 7.33 (d, *J* = 8.0 Hz, 2H, *p*-Tol), 7.39–7.50 (m, 2H, Ar), 7.53–7.59 (m, 2H, Ar, CH=), 7.79 (d, *J* = 8.3 Hz, 2H, *p*-Tol), 8.01 (dd, *J* = 7.6, 1.8 Hz, 1H, Ar). ^13^C NMR (100 MHz, acetone): δ 20.6, 96.0, 124.5, 125.5, 127.5, 129.6, 129.9, 130.3, 130.5, 133.3, 133.31, 134.1, 134.71, 140.3, 147.4, 170.3. Anal. calcd. for C_18_H_13_ClO_2_: C: 72.86%; H: 4.42%; Cl: 11.95%; found: C: 72.35%; H: 4.51%; Cl: 12.06%. 

A suitable single crystal of (**1**) was obtained by slowly cooling a hot 96% EtOH solution. The crystal was washed with cooled EtOH and dried in vacuum. The dimensions of the crystal were 0.30 × 0.05 × 0.05 mm^3^.

### 3.3. Crystal Structure Determinations and Refinement

An X-ray diffraction study of (**1**) was performed on a Bruker SMART APEX II CCD diffractometer (Cu*K*α radiation, graphite monochromator, ω-scan) at 100 K. Empirical absorption corrections were made using the SADABS program [44,45,46,47]. The structure was solved by a direct method and was refined by full-matrix least-squares versus *F*^2^_hkl_ with anisotropic displacement parameters for all non-hydrogen atoms. The hydrogen atoms were placed in the calculated positions and were refined geometrically by using a riding model with *U*_iso_(H) = 1.2*U*_eq_(C) and *U*_iso_(H) = 1.5*U*_eq_(C) for methyl groups. 

The refinement was performed with the SHELX SHELXL-2014/7 software package version [48]. The structure was refined as an inversion twin (batch scale factor, BASF = 0.36). The crystal data, data collection, and structure refinement details are summarized in Table 4. The packing diagram and parameters of non-covalent interactions were obtained using Mercury 3.0 software [49].

### 3.4. DFT Calculations

The coordinates from the X-ray data were used as the initial coordinates. All structures were fully optimized by using tight convergence criteria and Becke’s three-parameter hybrid functional combined with the Lee–Yang–Parr correlation functional (B3LYP [50,51,52]) with the 6-311++G(d,p) basis set. The optimized coordinates of each of the two forms were used separately for relaxed scans of the C(2)–C(5)–C(6)–C(7)/C(2A)–C(5A)–C(6A)–C(7A) torsion angle with an increment of 10°. The energy profiles of the rotation processes were analyzed for extrema to evaluate the rotational barrier energy.

## 4. Conclusions

The two crystallographically independent forms for (**1**) have similar geometries but are not superimposable. Thus, while the flat furan-2(3*H*)-one parts are superimposable, the mean planes of the terminal and substituted phenyl groups are rotated to different extents with respect to the plane of the furan ring in the two. 

The quantum-chemical modeling of two non-flat forms (**1a**) and (**1b**) found in the single crystal of arylmethylidene derivative of furan-2(3*H*)-one (**1**) describes them well as rotamers by the presence of two almost energetically equivalent global minima on the energy curve as a function of the C(2)–C(5)–C(6)–C(7)/C(2A)–C(5A)–C(6A)–C(7A) dihedral angle. The close H···H and H···Cl contacts in rotamers of (**1**) are the reason for their non-planar structures. According to the quantum chemical calculations, the energy curve for the rotation along the single C(5)‒C(6)/C(5A)‒C(6A) bond of each rotamer reveals two unequal rotational barriers of 5.66 kcal/mol and 0.67 kcal/mol. That each rotamer can easily transform to the other during the rotation by 50° is the cause for the co-crystallization of these rotamers.

## Figures and Tables

**Table 1:** Parameters of the stacking interactions in the single crystal of (**1a**); (**1b**).

Ring	Interplanar Distance, Å	Intercentroid Distance, Å	Ring Offset, Å	Angle θ, °
**1a**				
*o*-Cl-Ar	3.470(3)	3.8037(2)	1.559(7)	24.19(5)
Fur-2(3*H*)-one	3.478(4)	3.8037(2)	1.540(8)	23.89(3)
*p*-Tol	3.380(3)	3.8037(2)	1.745(7)	27.31(4)
**1b**				
*o*-Cl-Ar	3.519(3)	3.8037(2)	1.444(7)	22.33(8)
Fur-2(3*H*)-one	3.453(4)	3.8037(2)	1.596(8)	24.81(8)
*p*-Tol	3.464(3)	3.8037(2)	1.572(7)	24.41(9)

**Table 2:** Important contacts (Å) in the extrema of the energy rotational curves of rotamers (**1a**) and (**1b**).

Extremum	Contact		C(5)H–Cl(1)		C(3)H–C(11)H		C(5)H–C(11)H		C(3)H–Cl(1)	
	Angle		1a	1b	1a	1b	1a	1b	1a	1b
Global min	−152.303	−147.749	2.690	2.722	2.242	2.307	3.711	3.692	5.657	5.583
Local max	−82.303	−87.749	3.647	3.535	4.095	3.899	3.014	3.121	3.776	3.995
Local min	−52.303	−47.749	4.065	4.105	4.852	4.932	2.536	2.483	2.943	2.881
Global max *	−2.303	2.2511	4.409	4.409	5.551	5.551	2.070	2.070	2.502	2.502
Local min	47.697	52.251	4.105	4.065	4.932	4.853	2.483	2.535	2.880	2.942
Local max	87.697	82.2509	3.536	3.648	3.900	4.097	3.120	3.013	3.994	3.774
Global min	147.697	152.251	2.723	2.690	2.308	2.243	3.692	3.711	5.581	5.655
Local max **	177.697	−177.749	2.543	2.543	1.968	1.968	3.807	3.807	6.009	6.010

* major barrier; ** minor barrier.

**Table 3:** Comparison of some important geometrical parameters (bonds and interatomic distances (Å), and angles and torsions (°)) of the rotamers from the X-ray study and calculations of each rotamer separately.

Geometrical Parameter	Experimental		Calculated	
	1a	1b	1a	1b
C(2)–C(5)	1.354(4)	1.351(5)	1.356	1.356
C(2)–C(3)	1.437(5)	1.447(5)	1.442	1.442
C(5)–C(6)	1.461(5)	1.452(4)	1.457	1.457
O(2)···C(5)H	2.714(4)	2.628(4)	2.629	2.629
C(5)H···Cl(1)	2.785(3)	2.651(3)	2.690	2.690
C(3)H···C(11)H	2.303(5)	2.151(5)	2.242	2.243
C(2)–C(5)–C(6)	124.4(3)	128.4(3)	127.858	127.843
C(2)–C(5)–C(6)–C(7)	−144.5(3)	157.6(4)	−152.303	152.251

**Table 4:** Experimental details.

Crystal data	
Chemical formula	C_18_H_13_ClO_2_
*M*r	296.73
Crystal system, space group	Orthorhombic, *Pna*21
Temperature (K)	100
*a, b, c* (Å)	28.2038(14), 25.8791(14), 3.8037(2)
*V* (Å^3^)	2776.3(3)
*Z*	8
*Z′*	2
Radiation type	Cu*K*α
µ (mm^−1^)	2.44
Crystal size (mm)	0.30 × 0.05 × 0.05
Data collection	
Diffractometer	Bruker *APEX* II CCD (charge-coupled device) area detector’ diffractometer
Absorption correction	Multi-scan *SADABS*
No. of measured, independent, and observed [*I* > 2σ(*I*)] reflections	26,357, 4925, 4084
*R*_int_	0.083
(sin θ/λ)_max_ (Å^−1^)	0.618
Refinement	
*R*[*F*^2^ > 2σ(*F*^2^)], *wR*(F^2^), *S*	0.051, 0.119, 1.00
No. of reflections	4925
No. of parameters	382
No. of restraints	1
H-atom treatment	H-atom parameters constrained
Δρ_max_, Δρ_min_ (e Å^−3^)	0.24, −0.42
Absolute structure	Refined as an inversion twin
Absolute structure parameter	0.36(2)

## Data Availability

The data presented in this study are available on request from the corresponding author.

## Sample Availability

Sample of the compound (**1**) is available from the authors.
